# Metals and Metalloid Concentrations in Fish, Its Spatial Distribution in PPC, Philippines and the Attributable Risks

**DOI:** 10.3390/toxics11070621

**Published:** 2023-07-18

**Authors:** Delia B. Senoro, Maria Mojena G. Plasus, Alejandro Felipe B. Gorospe, Ronnel C. Nolos, Allaine T. Baaco, Chitsan Lin

**Affiliations:** 1 School of Civil, Environmental and Geological Engineering, Mapua University, Manila 1002, Philippines; 2 Resiliency and Sustainable Development Laboratory, Yuchengco Innovation Center, Mapua University, Manila 1002, Philippines; afbgorospe@mapua.edu.ph; 3 Mapua-MSC Joint Research Laboratory, Marinduque State College, Boac 4900, Philippines; nolos.ronnel@mscmarinduque.edu.ph; 4 College of Fisheries and Aquatic Sciences, Abba Building, Western Philippines University, San Juan 5300, Philippines; mojena.plasus@wpu.edu.ph (M.M.G.P.);; 5 College of Environmental Studies, Marinduque State College, Boac 4900, Philippines; 6 College of Agriculture, Forestry and Environmental Sciences, Western Philippines University, San Juan 5302, Philippines; 7 Department of Marine Environmental Engineering, National Kaohsiung University of Science and Technology, Kaohsiung 81157, Taiwan

**Keywords:** brackish, carcinogenic, fish, health risk, marine, metals, XRF

## Abstract

Fish is an important source of protein in human meals around the world. However, the fish that we are eating may be contaminated with toxicants such as metals and metalloids (MMs), which may pose health risks to consumers. Information on MMs content in fishes and their potential spatial distribution scenarios would provide knowledge to the community to create strategies and protect human health. Hence, this study assessed and determined the health risk levels of MMs in both brackish and marine water fish (BMF) in Puerto Princesa City (PPC), Palawan Province, Philippines. PPC has an existing abandoned open mine pit near the PPC coastline called the “pit lake”. The concentrations of As, Ba, Cu, Fe, Mn, Hg, and Zn in fishes were analyzed using portable Olympus Vanta X-ray Fluorescence (pXRF), and the spatial distribution of MMs concentrations in BMF was analyzed using a GIS (geographic information system). Fishes were sampled from fishing boat landing sites and nearby seafood markets. The results revealed that the concentration of MMs in marine fish was generally higher than the brackish water fish. It was recorded that the Hg concentration in marine water fish meat was higher than in brackish water fish meat. The Mn concentration in marine water fish exceeded the permissible limits set by international bodies. An elevated concentration of Mn in BMF was detected across the northern part of PPC, and an elevated concentration of Hg in marine fishes was recorded in the southeast area, where the fish landing sites are located. Ba was also detected in BMF across the southern part of PPC. Moreover, an elevated concentration of Cu was detected in MBF in the northeast and in marine fish in the southeastern area of PPC. Further, this paper elaborates the non-carcinogenic and carcinogenic risks of these fishes to the PPC population and tourists with respect to the MMs content in fish meat.

## 1. Introduction

Fish contains high-quality proteins, polyunsaturated fatty acids, vitamins, and minerals and is an essential source of healthy food throughout the world [1,2]. Recently, however, growing pollution and toxic contamination have caused a decline in the catch and consumption of fish both from the marine and freshwater ecosystems [3,4].

Heavy metals can usually be found in really low concentrations and are essential components of the aquatic environment [5]. However, heavy metals can be accumulated in the body of fish and other aquatic organisms through ingestion or by passing through semi-permeable membranes [6,7,8,9], such as fish skin. The consumption of fish contaminated with toxic metals shows several adverse effects [2,10]. Many factors such as size, sex, reproductive cycle, feeding habits, and swimming patterns are affected by the quality of living environment and have a role in the bioaccumulation of metals in fish [11,12]. Metals such as copper (Cu) and zinc (Zn) are essential for fish metabolism, while others such as mercury (Hg), cadmium (Cd), and lead (Pb) have no known role in biological systems [13,14]. Consumption of fish both from marine and freshwater environments contaminated with metals can cause health problems such as impaired renal and liver function, decreased cognitive function, impaired reproductive capacity, hypertension, neurological changes, and cancers [15,16,17,18].

Puerto Princesa City (PPC) is the capital city of Palawan Province in the Philippines. It is an island that has beautiful scenery, stunning islets, and is known for its clean and natural environment [19]. PPC is one of the tourist capitals of the Philippines and the main supplier of marine fish [20]. Based on the tourism office report, 1,170,083 people visited PPC in 2019, but this was reduced to 156,501 people in 2020 due to the pandemic [21]. Local fisherfolk in the Philippines often choose to directly sell fish, particularly grouper, to restaurant owners and/or tourists instead of to middlemen to deliver fresh fish, maximize their profit, and directly serve tourists [22]. Thus, it is important to monitor the fish quality being consumed by tourists and the local population, as PPC is known for its fresh marine fishes.

PPC as a tourism city has been challenged by industrialization. The presence of mining sites within and outside the city has been helpful to the local economy; however, environmental quality control and monitoring have become a challenge due to the lack of qualified laboratories, competent personnel, and funding. At present, based on a report by the Local Disaster Risk Reduction and Management Office of PPC, there are at least ten complex hazards in PPC related to heavy metals. These hazards include Hg contamination, water resource contamination, and chemical poisoning [20].

One of the mining companies operated Hg mining activities in Barangay Tagburos, PPC, from 1953 to 1976 through an open pit mining technique. This is a coastal barangay of Honda Bay in PPC. The Hg open mining site was abandoned after its operation in 1976. Currently, the abandoned Hg open mining pit still exists, which the local population calls “pit lake”. Remediation has been recommended by the government because of its possible adverse effect on the health of the people surrounding the abandoned open mine pit; however, the necessary remediation remains a recommendation document as of the writing of this scientific paper. One of the fishing grounds of Honda Bay has a designated fishing port (fishing boat landing site) and fish market near Brgy. Tagburos, where high mercury contamination has been observed [20,23,24]. Further, the effluents from the city pass near PPC Bay, which is also near the culture site for milkfish and other cage-farmed fish commodities [20]. This scenario calls for a comprehensive assessment of the marine water and fish meat quality.

The PPC Local Government is implementing a water quality monitoring system particularly to monitor algal bloom. However, attention has not been given to the monitoring of metal and metalloid (MMs) concentrations in the water and the fish being consumed by the PPC population and its tourists. In addition, no studies have been published on the bioaccumulation of MMs in brackish water and marine fish specific to PPC. Hence, an assessment of the brackish and marine fish meat quality was carried out to assist the PPC local government to create monitoring strategies and appropriate interventions to protect the health of its constituents, tourists, and its local economy.

## 2. Materials and Methods

### 2.1. Study Site and Sampling Locations

The study was conducted in PPC, the capital city of Palawan Province, Philippines. PPC is located at 9°30′ N and 118°30′ E, with a population of 1.2 million, has 66 barangays, and is internationally known for its natural resources such as underground rivers, beautiful beaches, and delicious seafood.

The province has a Type III climate characterized by a short dry season and sporadic rainfall months. The dry season typically lasts from January to April, while the rest of the year experiences the rainy season, with September being the wettest month [25]. The annual precipitation is 1314 mm, and the rainy season records a monthly average precipitation of 185 mm [26], describing runoff events that potentially carry contaminants from a higher to a lower elevation.

### 2.2. Collection, Processing, and Detection of Metals and Metalloids (MMs) in Fish Samples

**Figure 1:**
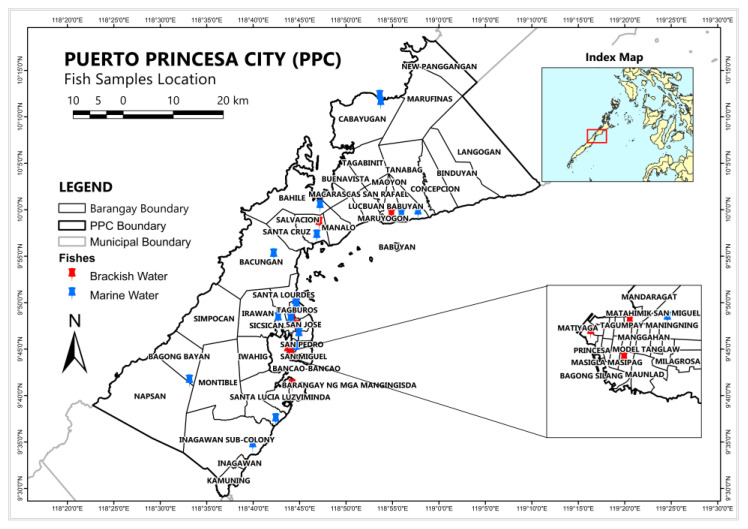
PPC and the 29 sampling sites of marine and brackish fish.

Fish samples were bought from the fishing boat landing sites and small markets, locally known as “talipapa”, of PPC towards the end of the rainy season. These fishing boat landing sites are for local trade. Twenty-nine sampling sites were recorded in various barangays of PPC, as shown in Table A1 and Figure 1. Five fish species were collected such as *Epinephelus coioides*, *Epinephelus* sp, *Cephalopholis* sp. (locally known as Lapua-Lapu, English name is grouper), *Rastrilliger kanagurta* (locally known as buraw), and *Chanos* (locally known as bangus, English name milkfish). These are the common fish types consumed by local residents. The fish samples collected from 29 sites, addressing a 95% level of confidence, comprised nine brackish water fish (*Chanos chanos*) and twenty marine fish (*Lujanus* sp., *Epinephelus coioides*, *Epinephelus* sp., *Cephalopholis* sp., and *Rastrilliger kanagurta*). The *Lujanus* sp., *Epinephelus* sp., and *Cephalopholis* sp. fishes are coral reef carnivore marine fishes; while *Rastrelliger* sp. is a pelagic marine water fish; *Chanos* fish thrive in both marine and brackish water. Carnivore fishes get most of their energy from a meat-based diet that could possibly mean eating some smaller fishes. Pelagic fishes live in water columns of the open seas, oceans, or lakes. Both *Chanos* and *Lujanus* sp. are omnivore fish. The *Chanos* fish samples in this study were collected from brackish water. The omnivore fish needs both meat- and plant-based for their food.

The EPA 823-B-00–007 protocol [27] was followed in handling and storing of the fish samples. The fish samples were washed with deionized water and placed in resealable plastic, labelled, arranged in a clean cooler, and brought to the laboratory for organization and MMs (Olympus Corporation of the Americas, Westborough, MA, USA) detection and analysis. No other complex preparation or pre-treatment is required. A portable Olympus Vanta portable X-ray Fluorescence (pXRF), (Olympus Corporation of the Americas, Westborough, MA, USA) analyzer was used for the detection and analysis of MMs in the fish samples. Hence, all fish samples were organized and analyzed within 24 h after actual collection. The calibration of the pXRF was carried out with the aid of the manufacturer before its use. The pXRF was calibrated using the Olympus Vanta blank in #2 zipper plastic bags, the Olympus Vanta XRF standard reference materials [28], and set to Geochem prior to the analysis of the fish samples [29,30]. The Olympus Vanta XRF is a handheld metal analyzer that provides rapid, accurate multi-elemental analysis and alloy identification, even during fieldwork. The limit of detection (LOD) for As, Ba, Cu, Fe, Mn, Hg, and Zn is 1, 5, 2, 12, 5, 1, and 1, respectively. The declared MMs concentration is the net concentration, i.e., after the background concentration of MMs was deducted.

### 2.3. Health Risk Assessment of MMs in Fish Samples from PPC

#### 2.3.1. Chronic Daily Intake

The chronic daily intake (*CDI*) of MMs through consuming brackish water and marine fish was calculated using Equation (1) [9].

(1) $$CDI=\frac{C_{i}\times E_{f}\times E_{d}\times IR\times C_{f}}{BW\times AT}\times 10^{-3}$$

where *C_i_* is the concentration of MMs in the fish samples (mg kg^−1^); *E_f_* is the exposure frequency (365 days y^−1^) [9]; *E_d_* is the exposure duration (69.39 years) [9]; *IR* is the ingestion rate of brackish water fish (7.23 g person^−1^ day^−1^) [31] and marine fish (11.62 g person^−1^ day^−1^) [31]; *C_f_* is the conversion factor (0.208) [9]; *BW* is the average body weight (60 kg) [9]; and *AT* is the averaging time ($E_{f}\times E_{d}$).

#### 2.3.2. Non-Carcinogenic Risk

The target hazard quotient (*THQ*) estimation approach used in the study provided estimates of the degree of non-carcinogenic health risk brought on by exposure to MMs in the fish [32]. The risks for the consumption of BMF were assessed based on Equation (2) [33]. As a general rule, when the *THQ* value is less than 1, it means the toxic effects of the specific MMs mentioned above are unlikely to occur. If the *THQ* is equal or greater than 1, it means there is a possible carcinogenic risk to the population. Therefore, appropriate intervention/s and protective measure/s should be made [32].

(2) $$THQ=\frac{CDI}{R_{f}D}$$

where *R_f_D* is the reference dose for the MMs (mg kg^−1^ day^−1^), as shown in Table 1. Moreover, the total target hazard quotient (*TTHQ*) was calculated following Equation (3) [34,35]. Summarizing *THQs* across MMs can act as a cautious assessment method to estimate high-end health risks rather than low-end risks. This is to safeguard the public from the potential adverse health consequences posed by several MMs [36].

(3) $$TTHQ=THQ_{AS}+THQ_{Ba}+THQ_{Cu}+THQ_{Fe}+THQ_{Mn}+THQ_{Hg}+THQ_{Zn}$$

It is inferred that the larger the value of *TTHQ*, the higher the probability of carcinogenic risk or health risks of toxic concerns [37].

**Table 1:** Reference dose (*R_f_D*) and slope factor (*SF*) of MMs.

MMs	*R_f_D* (mg kg^−1^ day^−1^)	*SF* (mg kg^−1^ day^−1^)	Reference
As	0.0023	1.5	[38,39]
Ba	0.2	-	[39]
Cu	0.037	-	[40]
Fe	0.7	-	[41]
Mn	0.14	-	[40]
Hg	0.00016	-	[36]
Zn	0.3	-	[40]

#### 2.3.3. Carcinogenic Risk

The lifelong risk of developing cancer as a result of exposure to a carcinogen(s) is known as carcinogenic risk (*CR*) [42]. Among the studied MMs in brackish water and marine fish, only As is categorized as a carcinogen by the International Agency for Research on Cancer (IARC) [43]. The *CR* was calculated following Equation (4) [42].

(4) CR=CDI×SF

where *CDI* is the chronic daily intake of MMs (mg kg^−1^ day^−1^ ) and *SF* is the slope factor (mg kg^−1^ day^−1^ ), as shown in Table 1 [38]. Cancer risk is categorized as negligible if *CR* < 1 × 10^−6^; acceptable if *CR* is within 1 × 10^−6^–1 × 10^−4^; high if *CR* is within 1 × 10^−3^–1 × 10^−1^; and very high if *CR* > 1 × 10^−1^ [44].

#### 2.3.4. Maximum Allowable Fish Consumption Rates

The maximum allowable fish consumption rates (*CR_lim_*) (g person^−1^ day^−1^) for both the non-carcinogenic and carcinogenic risks of MMs in brackish water and marine fish were calculated [45]. The *CR_lim_* for the non-carcinogenic and carcinogenic health risks of consuming fish contaminated with MMs are shown in Equations (5) and (6), respectively [46].

(5) $${\mathrm{CR}}_{lim}=\frac{R_{f}D\times BW}{C_{i}}$$

where *R_f_D* is the reference dose of MMs (mg kg^−1^ day^−1^), as shown in Table 1; BW is the average body weight for adults (60 kg) [9]; and *C_i_* is the concentration of MMs in fish (mg kg^−1^).

(6) $$CR_{lim}=\frac{ARL\times BW}{C_{i}\times SF}$$

where *ARL* is the acceptable lifetime risk level (1×10^−5^) [46] and *SF* is the slope factor (mg kg^−1^ day^−1^), as shown in Table 1. Just like in the *CR* calculation, only the As in fish was calculated using Equation 6 as it is the only identified carcinogen [43]. Generally, when the *CR_lim_* exceeds the determined average daily consumption of fish [31], the food does not present non-carcinogenic and carcinogenic health concerns.

### 2.4. Statistical Analysis

The descriptive statistics of the mean concentration of MMs in brackish water and marine fish were calculated using Excel software version 16.0.5332.1000 (Redmond, WA, USA). A Pearson rank correlation matrix coupled with a correlogram was also calculated using RStudio version 1.4.1106. Additionally, IBM SPSS Statistics version 23.0.0.0 was used in performing the Kruskal–Wallis test and hierarchical cluster analysis (HCA) to identify significant differences and homogenous clusters across the MMs in brackish water and marine fish [47,48]. In order to evaluate how cohesive, the clusters generated were, a dendrogram was also created, in which correlations between the various components are clearly visible [49].

### 2.5. Spatial Distribution Maps of MMs in PPC

The spatial distribution of MMs in the brackish water and marine fish of PPC was mapped using the Geographic Information System (GIS), ArcGIS Desktop 10.8.1 ArcPro2.8 [50].

#### The Inverse Distance Weighting

Raster data for the spatial distribution of MMs concentration was derived from the Inverse Distance Weighting (IDW) method of spatial interpolation using the IDW tool in ArcGIS Desktop. The collected sample points for fishes in the study area were used as the input in the IDW tool to generate raster data that showed the spatial distribution of MMs concentrations in fishes within the study area.

The IDW technique is a deterministic type of spatial interpolation that assumes objects closer to one another, i.e., within a certain radius, are more similar than those objects that are further apart [51]. Weights assigned to sample points are heavier or higher when they are closer to an estimated value point. This is raised to a specific power or exponent [52], shown as Equation (7).

(7) Zj^=∑izidinj∑i1dinj

where $\hat{Z_{j}}$ is the estimated value of unsampled point *j*, *Z_i_* is the value of sample point *i*, *d_ij_* is the distance from point *i* to *j*, and *n* is the weight parameter applied as an exponent to distance *d_ij_*. This implies that the larger the value of *n*, the greater influence has the sampled point *i* compared to the unsampled point *j* [53].

## 3. Results

### 3.1. Heavy Metals and Metalloids in Fish of PPC

The Olympus Vanta XRF is a rapid multi-element and alloy analyzer. It only requires washing of the fish, placing it inside the resealable plastic, and proper labelling. Hence, it detects various metals and alloys within its limit of detection simultaneously. Results of metals analysis by XRF showed no concentration of Cd, Ni, and Pb detected. However, it recorded the presence of Ba, Cu, Fe, Hg, and Zn. The range of concentrations of these various MMs in fish and its comparison to the permissible limit [9,54,55,56] is presented in Table 2. It is shown in the Table that, in general, except for Zn and Ba, the MMs concentration in marine fish was higher than the brackish water fish. The highest MMs concentration in brackish water fish was Zn at 14.118 mg kg^−1,^ while in marine water fish was Fe at 11.630 mg kg^−1^. It was recorded that Mn in both marine and brackish water fish exceeded the permissible limits. Additionally, the concentration of Hg in marine fish was almost near the permissible limit set by the European Commission (EC) [54]. Other MMs in BMF, such as Ba, Cu, Fe, and Zn, did not exceed the permissible limits set by FAO/WHO. All the As and Hg concentration in brackish water fish was below the limit of detection (LOD). The trend of MMs concentrations in brackish water fish and marine water fish were in the following order: Zn > Fe > Mn > Cu > Ba > Hg > As and Fe > Zn > Cu > Mn > Ba > Hg > As, respectively. The results of the Kruskal–Wallis test showed that the MMs across BMFs originated from the same distribution due to its record of 5% significant differences (Table A2).

### 3.2. Spatial Distribution of MMs in the Fish of PPC

**Figure 2:**
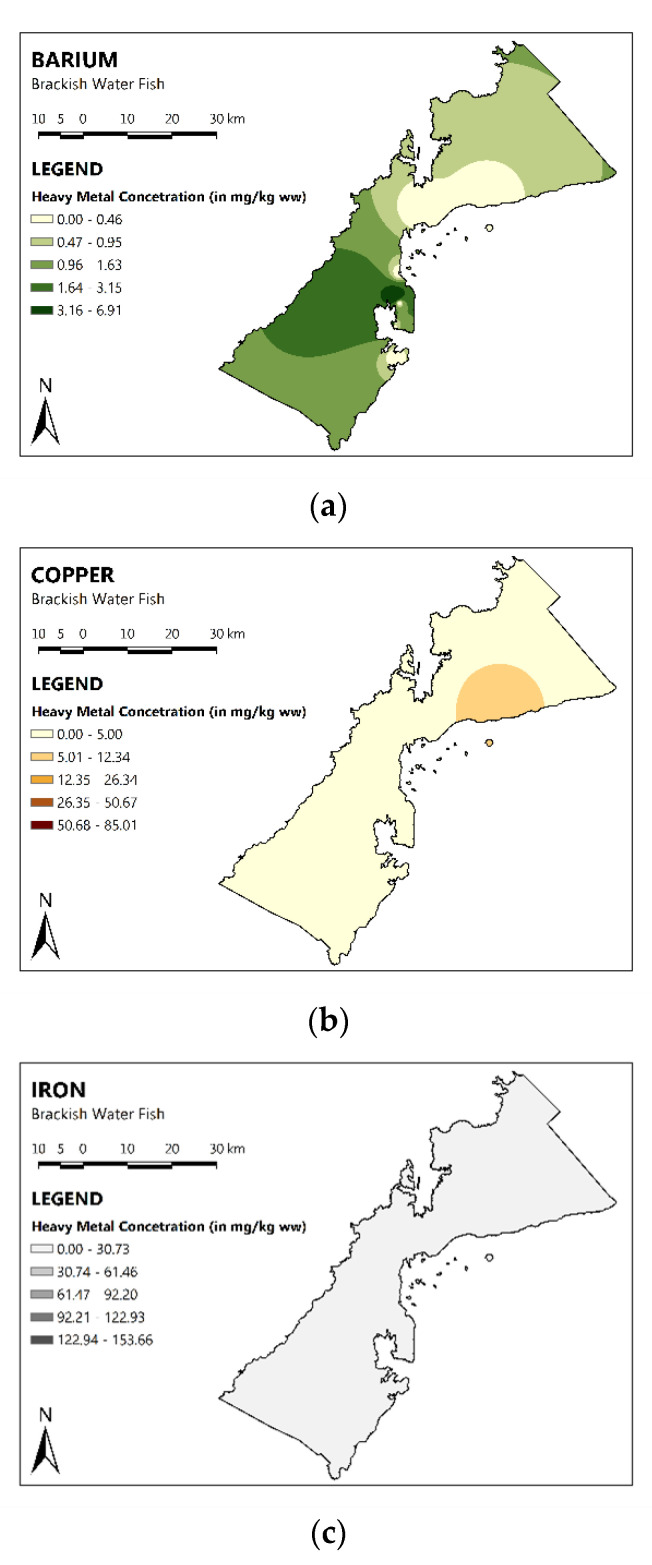

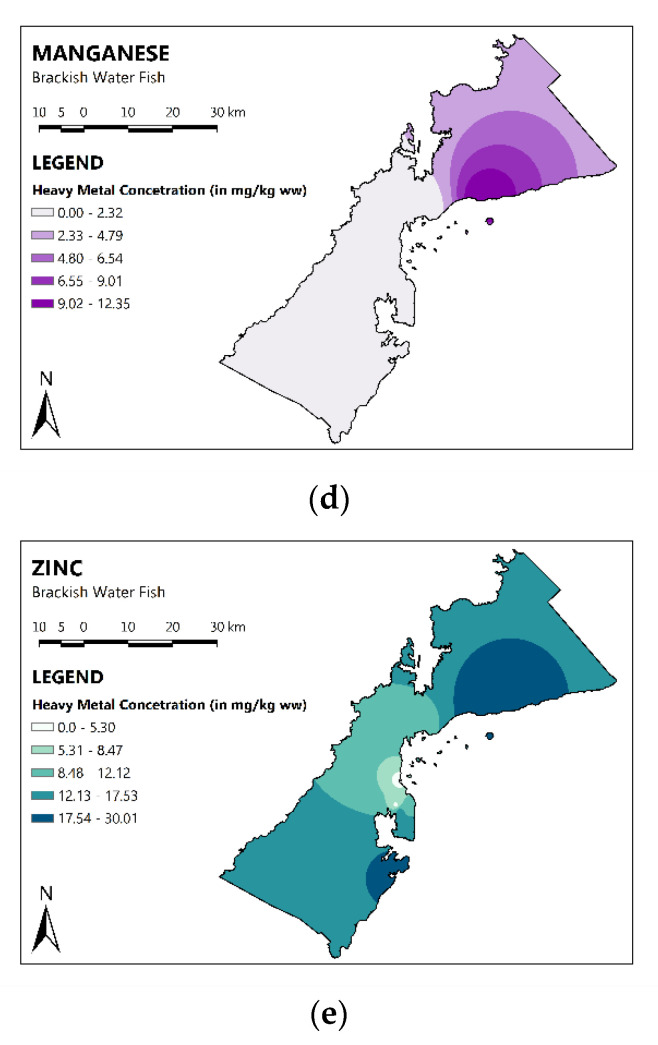
Spatial distribution of MMs in brackish water fish. The darker the color, the higher the MMs concentration.

The spatial distribution maps of MMs in the brackish water fish of PPC are shown in Figure 2. The recorded concentrations of Cu, Mn, and Zn were found to be the highest in the northeastern part of PPC. While the concentration of Ba was highest in the southwestern part of PPC. The concentration of Fe was similarly distributed all over PPC and did not illustrate specific area of concern. There were no spatial distribution maps for the As and Hg, as the concentrations of these MMs were below the detection limit.

**Figure 3:**
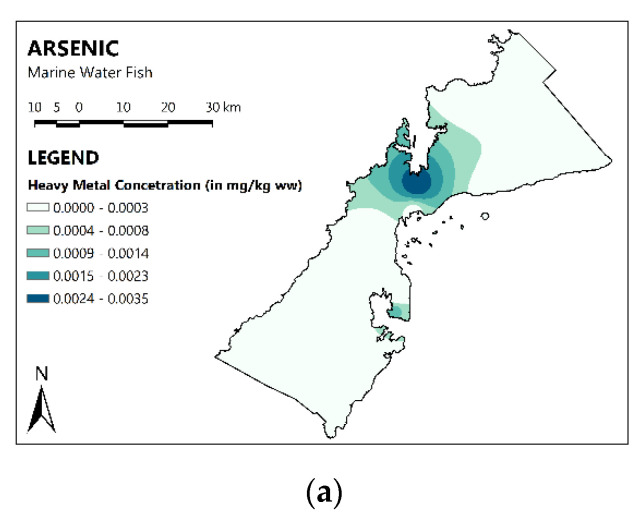

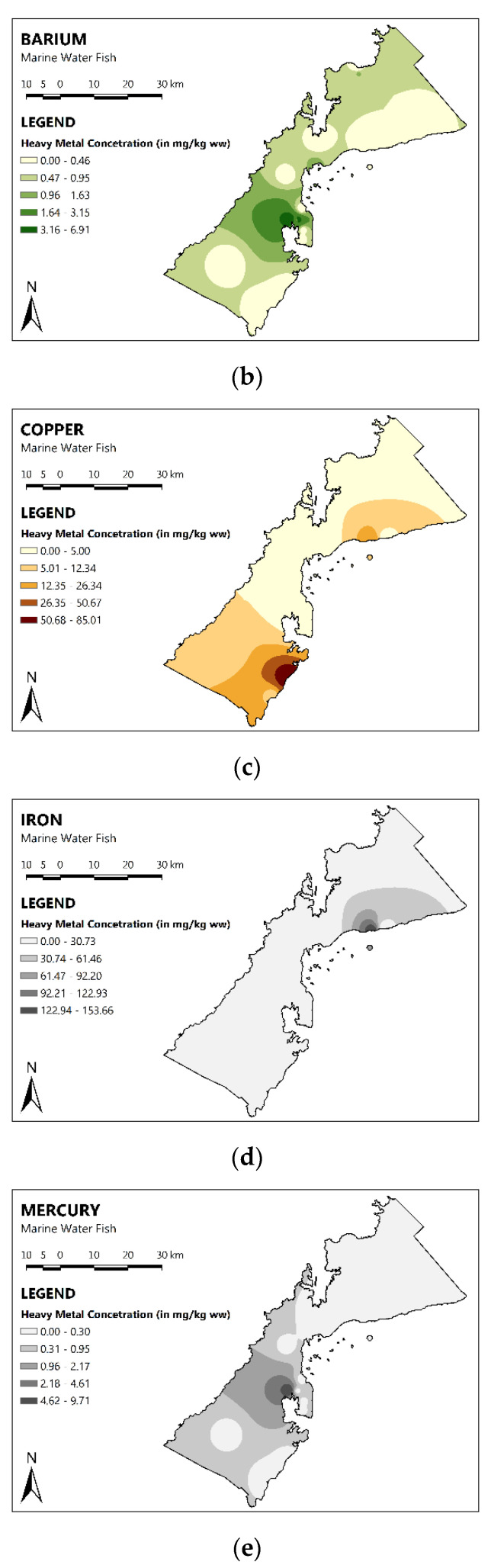

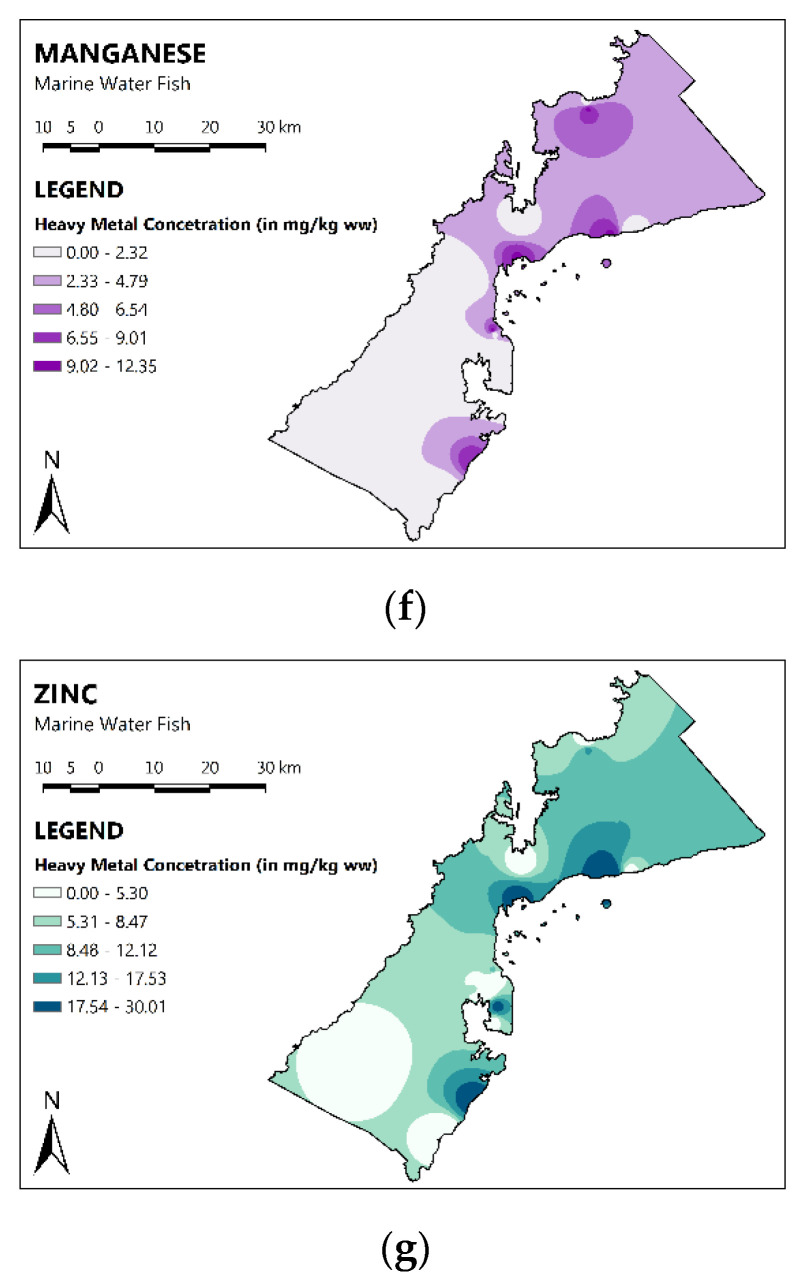
Spatial distribution of MMs in marine water fish. The darker the color, the higher the MMs concentration.

Additionally, the spatial distribution maps of MMs in the marine fish of PPC are shown in Figure 3. The concentrations of Ba, Cu, and Hg were highest in the southwestern part of PPC; As was highest in the northwestern part of PPC; and the concentrations of Mn and Zn were highest in major parts of PPC.

### 3.3. Health Risk Assessment of MMs in Fish

**Figure 4:**
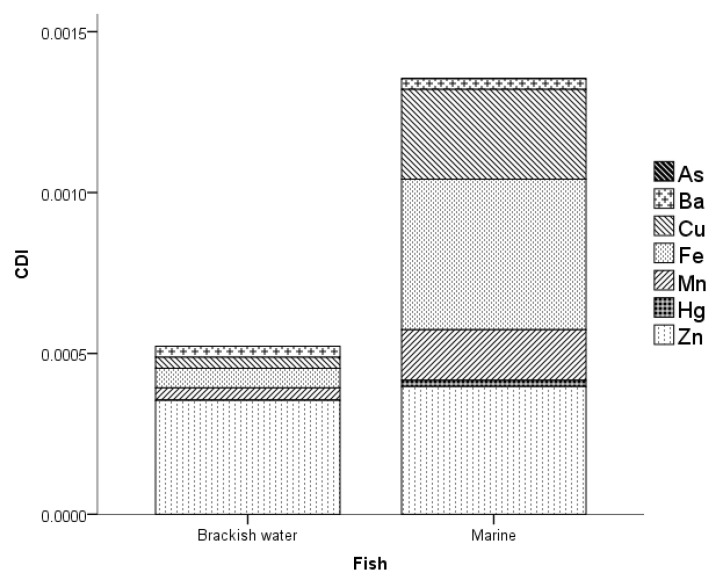
*CDI* of MMs in the fish of PPC.

The chronic daily intake (*CDI*) of MMs in fish in PPC is shown in Figure 4. The computed *CDI* of MMs in brackish water fish ranged from $0\;\mathrm{to}\;3.54\times 10^{-4}$. The concentration of Zn contributed significantly to the total *CDI* of MMs in brackish water fish accounting for 67.77%. Additionally, the computed *CDI* of MMs in marine water fish ranged from $9.21\times 10^{-9}\;\mathrm{to}\;4.68\times 10^{-4}$. On the other hand, the Fe contributed largely to the total *CDI* of MMs in marine water fish, which was equivalent to 34.58%. The trend of *CDI* in BMF was in the following order: Zn > Fe > Mn > Cu > Ba > Hg > As and Fe > Zn > Cu > Mn > Ba > Hg > As for brackish and marine water fish, respectively.

**Figure 5:**
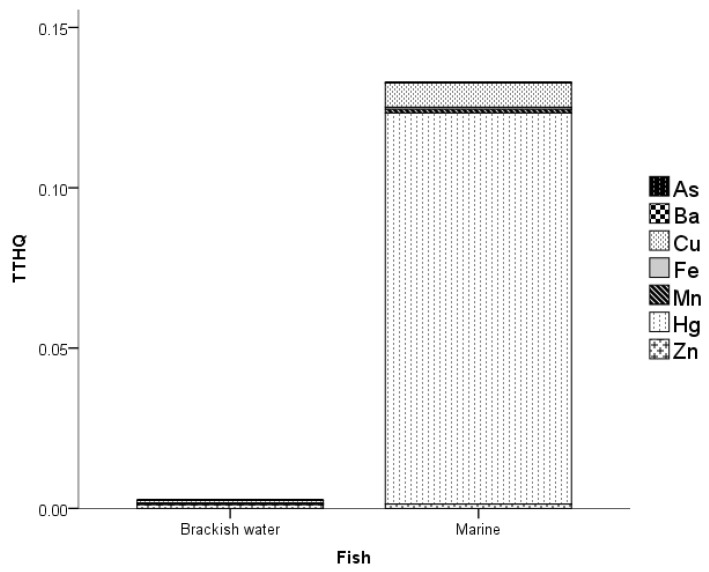
*TTHQ* of MMs in the fish of PPC.

The total target hazard quotient (*TTHQ*) of MMs in the BMF in PPC is shown in Figure 5. It can be observed from the Figure that the *TTHQ* of MMs in marine fish is relatively greater than the brackish water fish recording about 96%. The *TTHQ* of MMs in brackish water fish ranged from $0\;\mathrm{to}\;1.18\times 10^{-3}$, while marine water fish recorded a *TTHQ* range of 3.07 × 10^−5^–1.22 × 10^−1^. The Zn and Cu contributed 44.6% and 35.2%, respectively, to the total *TTHQ* in brackish water fish; both accounted for almost 80%. Further, it is observed from the Figure that Hg contributed largely to the total *TTHQ* in marine fish, accounting for more than 90%. This was followed by Cu, which accounted for 5.7% of the total *TTHQ* in marine fish. Both the *THQs* of all MMs in BMF did not exceed the threshold 1, which indicates that toxic effects are unlikely to occur [32,34,55,56,57] by consuming BMF in PPC. The trends of *TTHQ* in brackish water and marine fish were on the following order: Zn > Cu > Mn > Ba > Fe > Hg > As, and Hg > Cu > Zn > Mn > Fe > Ba > As, respectively.

To assess the carcinogenic risk of consuming fish contaminated with MMs, the carcinogenic risk (*CR*) was calculated, as shown in Table 3. Only As was included in the *CR* calculation as it was the only MM in this study that was identified by the IARC as a carcinogen [58]. It was shown in the Table that the *CR* of the brackish water fish was 0, which was lower than the threshold value of $1\times 10^{-6}$ indicating a negligible risk of developing cancer [42,44]. The *CR* of the marine water fish was $1.38\times 10^{-8}$ which was also lower than the threshold value of $1\times 10^{-6}$ indicating negligible risk of developing cancer [42,44] from the consumption of marine water fish.

Table 4 shows the maximum allowable fish consumption rates (*CR_lim_*) that a 60 kg adult can consume in a day. It was recorded that all the *CR_lim_* (carcinogenic risk limit) of BWF were higher than the average daily consumption of brackish water fish (7.23 g person^−1^ day^−1^) and marine water fish (11.62 g person^−1^ day^−1^) [31]. This indicates that the fish investigated in this specific study did not pose carcinogenic health risks to the local population [59,60]. The *CR_lim_* for the non-carcinogenic health risks of consuming brackish water fish ranged from 1619.06 to 17,219.82 g person^−1^ day^−1^ while marine fish ranged from 19.77 to 14,629.68 g person^−1^ day^−1^. The lowest *CR_lim_* of consuming fish was recorded in marine fish with Hg content which was equivalent to 19.77 g person^−1^ day^−1^. It was 8.15 g higher than the average daily consumption, which indicated that if the local population increases their consumption than the recorded *CR_lim_*, the potentially toxic effects that are negative to health may occur.

Moreover, for the carcinogenic health risks of consuming marine fish contaminated with As, the *CR_lim_* was approximately 115 times higher than the average daily consumption (11. 62 g person^−1^ day^−1^) [31]; this also indicates that the carcinogenic risk posed by consuming marine fish contaminated with As by the tourists and local population was very low [61].

### 3.4. Relationship of MMs in Brackish Water and Marine Water Fish

**Figure 6:**
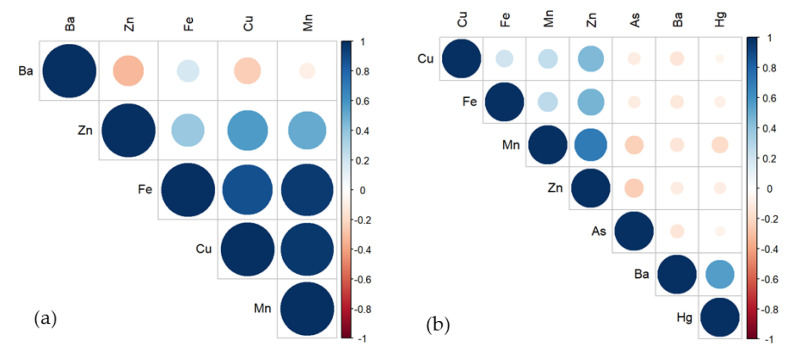
Correlation of MMs in (**a**) brackish water and (**b**) marine fish.

The correlograms that show the correlation between MMs in fish are shown in Figure 6. As and Hg were not included in the correlation analysis of MMs in brackish water fish as all the data observed were below LOD. Figure 6a shows that high to very high significant positive correlations existed between Fe–Cu (r = 0.874, *p* = 0.002); Cu–Mn (r = 0.968, *p* = 0); and Fe–Mn (r = 0.950, *p* = 0) at 1% significance difference level (2-tailed). Moreover, Figure 6b also shows medium to high significant positive correlation between Mn–Zn (r = 0.704, *p* = 0.001) at 1% significance difference level (2-tailed) and between Ba–Hg (r = 0.550, *p* = 0.012) and Fe–Zn (r = 0.465, *p* = 0.039) at 5% significance difference level (2-tailed).

**Figure 7:**
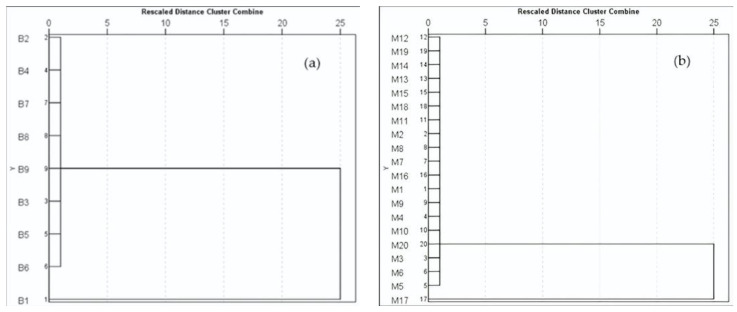
Dendrograms classifying the (**a**) brackish water fish and (**b**) marine fish based on *THQ*.

The hierarchical cluster analysis (HCA) of brackish water and marine fish based on *THQ* [62] was represented with dendrograms (Figure 7). In Figure 7A, two (2) clusters were classified. The first cluster comprised eight (8) brackish water fish samples (B2, B4, B7, B8, B9, B3, B5, and B6), approximately 88.89%, deemed safe for consumption. On the other hand, the second cluster was comprised of one (1) brackish water fish sample (B1), approximately 11.11%, which was recorded to have the highest *THQ* among the brackish water fish investigated (Table A1).

Additionally, Figure 7B shows the dendrogram in marine fish based on *THQ,* which has two (2) clusters. The first cluster was comprised of nineteen (19) marine fish samples (M12, M19, M14, M13, M15, M18, M11, M2, M8, M7, M16, M1, M9, M4, M10, M20, M3, M6, and M5), approximately 95%, which were deemed safe for consumption. The second cluster, on the other hand, was comprised of one (1) marine fish sample (M17), approximately 5%, which was found to have the highest *THQ* among the group and exceeded the threshold value for *THQ*. This indicates that marine fish sample M17 may be unsafe for consumption (please see Table A1). Generally, both dendrograms for BMF based on *THQ* revealed two (2) clusters.

## 4. Discussion

The consumption of fish is essential for human health and growth because of its nutritional content. However, pollutants such as MMs carried by runoff from abandoned mine pits, pit lakes, and industrial, uncontrolled discharges found their way to the aquatic environment and were eventually consumed by fishes. These pollutants can be ingested by aquatic organisms and eventually enter the food chain [63,64]. These MMs can bioaccumulate along the food chain where aquatic organisms in the higher trophic level, such as fish, have higher MMs content. This poses human health risks to the population who consumed such fish contaminated with MMs [65]. The determination of MMs levels in foods such as fish has gained important attention in recent years [9,32,66,67,68].

The findings of the study showed that among the MMs in BMF analyzed, it was Mn concentrations in both BMF were higher than the permissible limit. This is a similar result to the study of Ali et al. (2021) [69], in which Mn was one of the metals that tend to bioaccumulate highly in the muscle and liver of common carp (*Cyprinus carpio*) exposed to manganese sulphate and chromium chloride solution for 96 h. Mn can be present in aquatic environments due to natural causes (i.e., weathering of rocks) but primarily from anthropogenic activities such as mining [70] and domestic and industrial effluents [71]. A constant intake of fish highly contaminated with Mn may pose adverse health effects to the local population, such as neurodegenerative disorders [72], liver damage [73], and cardiovascular diseases [74]. In addition, the concentration of Hg in marine fish almost reached the permissible limit set by the European Commission (EC) [54]. 

Generally, wild fish are exposed to Hg^2+^ and methylmercury (MeHg) both from water and food. Hg has a strong affinity with elements Se and S which are mostly present as selenols and thiols in organisms like fish [75]. Cysteine (Cys) is the most abundant thiol in fish and the major complexing agent in the muscle of fish, which enhances the assimilation of MeHg from the environment [76]. This is one of the reasons why high accumulation of Hg in aquatic organisms such as fish frequently occurs. This finding was similar to the work of Nava et al., that showed Hg content in aquatic products was higher than land-based products [77]. The Hg pollution was recorded in the province of Palawan, Philippines, and was associated with the mining of cinnabar (HgS), known as the most common ore deposit of Hg [78].

**Figure A1:**
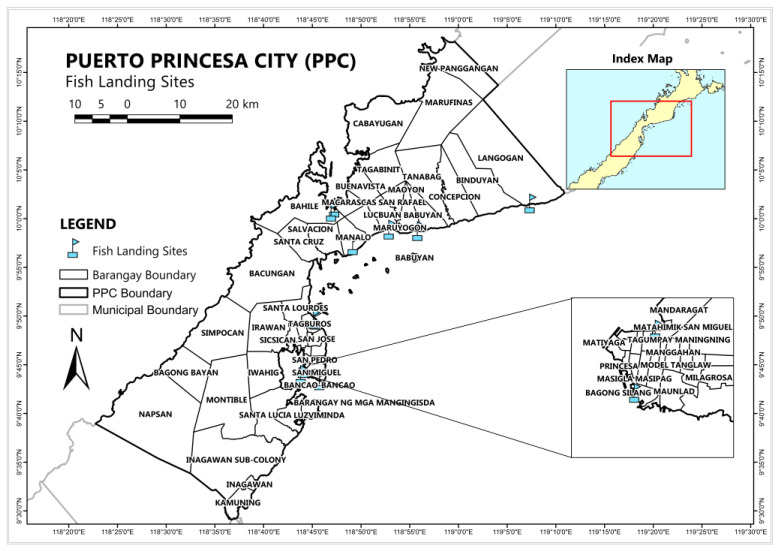
Fish landing sites in PPC, Palawan.

Further, the deposit also contains an abundant amount of pyrite (FeS_2_), which is hazardous because FeS_2_ is a mineral that produces acid-mine drainage (AMD) [78]. This AMD makes the water more acidic, which, in turn, hastens the solubility and reactivity of metals like Hg. This makes the Hg bioavailable for aquatic organisms like fish. Hg poisoning of residents near the abandoned mine site in PPC, Palawan was already reported due to exposure to mine tailings and ingestion of contaminated marine fish [79]. Symptoms such as nausea, vomiting, chest pains, palpitations, kidney dysfunction, and even death may manifest due to acute toxicity to Hg [80]. On the other hand, chronic exposure to Hg can cause cardiovascular and developmental toxicity, neurotoxicity, and immunotoxicity [81]. Exposure of pregnant women to MeHg also poses a severe impact on the neurodevelopment of new born babies [82]. These contaminants and their clinical manifestations to the local population shall be looked into by the local government and health units of PPC. This is to monitor possible cases of Hg intoxication and to create appropriate strategic program(s) to improve the environmental quality and the health of the tourists and population. Also, the fish landing sites (Figure A1) and the registered mining sites in PPC (Table A3) were recorded during the project study implementation. The detected MMs in fish samples, especially Hg in marine water fish, can be attributed to the presence of existing and abandoned open mining sites [83,84] (Table A3) near the fishing grounds and fishing ports at the southeast portion of PPC. Based on Figure 3, these are also the areas where elevated concentration of Hg was detected in BMF. Similar cases were recorded in several regions, such as the Pb concentrations, were found in *Epinephelus* sp. in fishes collected from Tuticorin, India [85], and Tanzania [86]. Concentrations of As and Hg were detected from *Epinephelus coioides* collected from the Persian Gulf [87]. Further, alarming concentrations of MMS were detected from *Rastrilliger kanagurta* samples collected from Visakhapatnam, India [88].

Additionally, climate variations are also important factors in the kinetics of toxic metals in aquatic environments. The primary negative impact of climate change on aquatic ecosystems and metal bioaccumulation is linked to the risks of the creation of new stress situations in which aquatic organisms are more susceptible to chronic intoxication [89]. The model simulations of Moe et al. [90] also showed that climate warming accelerates the cycling of toxic metals and metalloids in aquatic ecosystems and increases their toxic properties. The work of Panebianco et al. also suggested that the presence of some elements (i.e., MMs) in aquatic products may indicate the co-existence of other pollutants [91].

Among the MMs analyzed, Zn and Fe recorded the highest concentration in brackish water and marine fish, respectively. These were also the MMs, which contributed largely to the *CDI* in brackish water and marine fish. Zn is considered an essential metal for growth, but excess amounts can be hazardous to fish and those who consume the fish meat [92]. High accumulation of MMs, especially Zn in brackish water fish, is associated with anthropogenic contaminants originating from a wide range of sources, i.e., industrial activities, household, and agriculture [93], osmoregulation of fish exposed to different environments [94,95] and the presence of high level of Zn in natural food [96]. Additionally, Fe is also an essential metal for humans, especially for menstruating and pregnant women, where iron-deficiency anaemia is prevalent [97]. The recorded Fe concentration in marine fish was not greater than the permissible limit [9] and can provide the dietary need for Fe of an individual.

The computed *THQs* of all MMs were not also greater than 1, which indicates that non-carcinogenic health risks were unlikely to occur. However, the *TTHQ* of marine fish was far greater than brackish water fish. This was attributed to the high Hg concentration in marine fish than brackish water fish. Marine fish in PPC was expected to have a high concentration of Hg, as the waste from a cinnabar mine was deposited along the coast of Honda Bay, Palawan in 1995 [98]. The results of the CR also show that As in brackish water and marine fish have a negligible cancer risk to the population. However, it shall be kept in mind that the presence of some MMs could be associated with the co-existence of other pollutants [93] that are attributable to the seasonal variations and weather conditions (such as floods) affecting the spread of different pollutants in the environment. Additionally, the *CR_lim_* for both non-carcinogenic and carcinogenic health risks were all greater than the average daily consumption of brackish water and marine fish [31], indicating that the fish investigated do not pose health risks to a 60 kg adult, similar to the findings of Zhong et al., [99] and Han et al., [2]. However, it should be noted that the *CR_lim_* decreases when the amount of MM concentration increases and the body weight decreases.

The results of the correlation analysis also revealed that some positive relationships exist between MMs in both brackish water and marine fish, indicating changes in the same direction (i.e., when a MM increases, other MMs also increase) [100]. Similarly, this may also reveal common absorption sites of MMs in both brackish and marine fish, their interaction, and possible source(s) of pollution [34,101]. Ali et al. (2022) [102], also investigated toxic metals in commercial fishes from Bangladesh and found highly positive relationships between toxic metals, suggesting common sources and distribution patterns. A study of the water, sediments, and fish in Yemen for metal contamination also shows that the levels of metals in the fish are positively correlated with the levels of metals in the water and sediment [103]. On the contrary, studies show that the concentrations of MMs in fish have no direct relationship with the fish’s length and weight. Jiang et al. [104] investigated the concentrations of heavy metals such as As, Cd, Cr, Cu, Hg, Pb, and Zn in eighteen (18) fish species from Heilongjiang River, China, recorded no significant correlation between fish size and the concentrations of heavy metals, particularly Cd, Cr, Cu, Pb, and Zn. Similar observations were recorded by Cais et al. [105] that concentrations of As, Hg, and Zn in the muscle and gills of *P. vachelli* collected from the Yangtze River, China showed no significant correlations with the fish length. Likewise, the concentrations of Cu and Zn in *P. reticulata* collected in a stream in Indonesia did not depend on the fish’s body weight. The body concentrations of these metals are apparently regulated at certain concentrations [106].

Generally, the dendrograms show that almost 97% of all the investigated fish were considered safe for consumption by the local population. The findings of this study can be considered for future research in brackish water and marine fish in PPC to better understand the bioaccumulation and kinetics of pollutants, particularly MMs in fish as well as their risks to human health [102]. This study utilized portable XRF in analyzing MMs in fish, which can be used for regular in situ monitoring by the local government, as it provides real-time detection results [29,107] that are rapid and accurate, and does not require sophisticated sample preparation and/or pre-treatment. Similarly, XRF is also a powerful technique for analyzing MMs in fish which is cost-effective and drastically reduces analytical time [30]. Possible sources of MMs contamination in the area aside from mining should be looked into by the local government to come up with effective mitigation measures. Remediation measures are highly recommended, especially in the abandoned Hg mine site in PPC, where remediation work has not been carried out for the past four decades [83]. Moreover, regular monitoring of MMs and other possible contaminants in fishponds should be done [85] as it is doable where commercial fishes like milkfish (*Chanos chanos*) and tilapia (*Oreochromis niloticus*) are usually cultured.

## 5. Conclusions

This study investigated the concentrations of metals and metalloids (MMs) in brackish water and marine fish in PPC, Palawan, Philippines. Also, the associated health risks to the population were evaluated and determined. The MMs that were analyzed using portable Olympus Vanta XRF include As, Ba, Cu, Fe, Mn, Hg, and Zn. Results revealed that Mn in both brackish water and marine fish exceeded the permissible limit for safe consumption. Additionally, the Hg in marine fish was at an alarming level, as it is almost along the permissible limit. Other MMs, namely As, Ba, Cu, Fe, and Zn, did not exceed the permissible limit set by FAO. WHO and EC Both the *CDI* and *TTHQ* of MMs in marine fish were greater than the brackish water fish due to their different aquatic environment and degree of exposure to MMs. The *TTHQ* for both brackish water and marine fish, on the other hand, did not exceed the threshold value implying that toxic effects may not occur as health risks by consuming the BMF. Further, the *CR* due to As in BMF posed “negligible” carcinogenic risks to the population as the calculated *CRs* were below the threshold value set by IARC and USEPA. The calculated *CR_lim_* for both non-carcinogenic and carcinogenic risks also shows that the average daily consumption of BMF by an adult does not pose health risks. The pXRF is a practical device for MMs’ detection in BMF as it can provide rapid and accurate MMs concentration. More research on MMs’ concentration monitoring and its health risks for more fish species, and other toxic metals, such as Cd, Cr, Pb, and Ni that are deemed carcinogenic, are recommended. The Hg in marine fish should be routinely monitored as the recorded concentrations were quite alarming and MMs pose neurodegenerative disorders. Furthermore, and based on the result of this study, extensive research on land-based products in PPC is warranted to generate more data to ensure the food safety of the local population and tourists.

## Figures and Tables

**Table 2:** Range of MM concentrations (mg kg^−1^) in fish with permissible limits.

Fish	As	Ba	Cu	Fe	Mn	Hg	Zn
Brackish	<LOD	<LOD–6.91	<LOD–9.60	<LOD–11.21	<LOD–12.35	<LOD	3.83–26.18
Marine	<LOD	<LOD–6.68	<LOD–85.01	<LOD–153.66	0–12.35	<LOD–9.71	0–30.01
FAO/WHO [9,56]	-	-	30	100	1	-	100
JECFA [55]	0.002	-	-	-	-	-	-
EC [54]	-	-	-	-	-	0.5	-

Note: FAO/WHO—Food and Agriculture Organization/World Health Organization; JECFA–Joint FAO/WHO Expert Committee on Food Additives; EC—European Commission.

**Table 3:** The *CR* of MMs in the fish of PPC.

Fish	*CR*	Risk [44,56]
Brackish Water	0	negligible
Marine	$1.38\times 10^{-8}$	negligible

**Table 4:** Maximum allowable fish consumption rates (*CR_lim_*) (g person^−1^ day^−1^).

Fish	MMs	*CR_lim_* (Non-Carcinogenic)	*CR_lim_* (Carcinogenic)
Brackish Water Fish	As	-	-
	Ba	8862.21	-
	Cu	1619.06	-
	Fe	17,219.82	-
	Mn	5423.41	-
	Hg	-	-
	Zn	1274.99	-
Marine Water Fish	As	78,750.00	1750.00
	Ba	14,629.68	-
	Cu	320.61	-
	Fe	3611.47	-
	Mn	2152.15	-
	Hg	19.77	-
	Zn	1824.69	-

## Data Availability

Not applicable.

## Appendix A

**Table A1:** Fish samples and sampling locations in PPC.

Environment	Code	Barangay	Type of Fish	Scientific Name	English Name	Latitude	Longitude	Weight (g)	Feeding Habit
Brackish Water	B1	Babuyan	Bangus	*Chanos* sp.	Milkfish	9.98029	118.91462	-	Omnivore
	B2	Mangingisda	Bangus	*Chanos* sp.	Milkfish	9.67527	118.73655	283.4	Omnivore
	B3	San Jose	Bangus	*Chanos* sp.	Milkfish	9.78193	118.74316	308.6	Omnivore
	B4	Tagumpay	Bangus	*Chanos* sp.	Milkfish	9.7423	118.73625	240.6	Omnivore
	B5	Salvacion	Bangus	*Chanos* sp.	Milkfish	9.96691	118.78505	386.3	Omnivore
	B6	Tagburos	Bangus	*Chanos* sp.	Milkfish	9.84264	118.74381	351.5	Omnivore
	B7	Liwanag	Bangus	*Chanos* sp.	Milkfish	9.74032	118.22863	405	Omnivore
	B8	Masipag	Bangus	*Chanos* sp.	Milkfish	9.73514	118.73515	445.3	Omnivore
	B9	Sta. Monica	Bangus	*Chanos* sp.	Milkfish	9.79418	118.73571	311	Omnivore
Marine	M1	Cabayugan	Buraw	*Rastrelliger* sp	Mackerel	10.18221	118.89548	-	Carnivore
	M2	Cabayugan	Bisugo	*Nemipterus* sp.	Threadfin bream	10.18221	118.89548	-	Carnivore
	M3	Salvacion	Waling	*Gazza* sp.	slipmouths	9.94371	118.7808	-	Carnivore
	M4	Salvacion	Black Lapu-lapu	*Epinephelus* sp.	grouper	9.94371	118.7808	-	Omnivore
	M5	Inagawan Sub-Colony	Red Lapu-apu	*Epinephelus* sp.	grouper	9.61427	118.70711	-	Omnivore
	M6	Babuyan	Black Lapu-lapu	*Epinephelus* sp.	grouper	9.98001	118.9325	-	Omnivore
	M7	Tagburos	Lapu-lapu	*Epinephelus* sp.	grouper	9.82518	118.74225	-	Omnivore
	M8	Tagburos	Dugso	*Letrinus* sp.	Emperor fish	9.82518	118.74225	-	Carnivore
	M9	Napsan	Buraw	*Rastrelliger* sp	Mackerel	9.68359	118.55226	258.3	Carnivore
	M10	Inagawan	Buraw	*Rastrelliger* sp	Mackerel	9.56812	118.66583	401.8	Carnivore
	M11	Sta. Monica	Buraw	*Rastrelliger* sp	Mackerel	9.79375	118.73385	212.8	Carnivore
	M12	San Rafael	Lapu-lapu	*Epinephelus* sp.	grouper	9.98571	118.96236	219.8	Omnivore
	M13	Cabayugan	Lapu-lapu	*Epinephelus* sp.	grouper	10.19408	118.89374	176.3	Omnivore
	M14	Bahile	Lapu-lapu	*Epinephelus* sp.	grouper	9.99743	118.78659	456.4	Omnivore
	M15	Bacungan	Lapu-lapu	*Epinephelus* sp.	grouper	9.90981	118.70345	304.6	Omnivore
	M16	Tagburos	Lapu-lapu	*Epinephelus* sp.	grouper	9.82009	118.74429	266.1	Omnivore
	M17	Sicsican	Lapu-lapu	*Epinephelus* sp.	grouper	9.79559	118.71157	323.2	Omnivore
	M18	Sta. Monica	Lapu-lapu	*Epinephelus* sp.	grouper	9.79381	118.73435	388.3	Omnivore
	M19	San Miguel	Lapu-lapu	*Epinephelus* sp.	grouper	9.7431	118.74359	452	Omnivore
	M20	San Manuel	Lapu-lapu	*Epinephelus* sp.	grouper	9.76748	118.74872	411.2	Omnivore

**Table A2:** Kruskal-Wallis test of MMs across brackish water and marine fish in PPC.

Null Hypothesis	Sig.	Decision
The distribution of As is the same across the brackish water and marine fish	0.334	Retain the null hypothesis
The distribution of Ba is the same across the brackish water and marine fish	0.764	Retain the null hypothesis
The distribution of Cu is the same across the brackish water and marine fish	0.222	Retain the null hypothesis
The distribution of Fe is the same across the brackish water and marine fish	0.772	Retain the null hypothesis
The distribution of Mn is the same across the brackish water and marine fish	0.359	Retain the null hypothesis
The distribution of Hg is the same across the brackish water and marine fish	0.502	Retain the null hypothesis
The distribution of Zn is the same across the brackish water and marine fish	0.118	Retain the null hypothesis

The significance level is 0.05.

**Table A3:** Registered mining sites in PPC, Palawan.

Name	Commodity/Mine	Coordinates
Nickel Mining		
Birong Nickel Occurence	Ni	9.4173° N 118.1993° E
Brookes Point Nickel Occurence	Ni	8.9173° N 117.8827° E
Coral Bay Nickel Corp.	Ni	8.483828° N,117.4377063° E
Palawan Rio Tuba Nickel Laterite deposit	Ni	8.5007° N 117.3994° E
Long Point Nickel Deposit-Palawan Island	Ni	9.6506° N 118.3326° E
Ipilan Nickel Prospect	Ni	8.8507° N 117.8493° E
Rio Tuba Mine	Ni	8.5868° N 117.4049° E
Guintalungan Nickel Deposit	Ni	8.5549° N 117.3863° E
Isabela Nickel Deposit	Ni	9.1006° N 117.8993° E
Zinc Mining		
Balabac Copper mine	Cu, Zn	7.9841° N 117.0660° E
Manganese Mining		
Pina–Balitbitin Manganese Occurence	Mn	12.0837° N 120.1993° E
Binabaan Manganese Occurence	Mn	11.731° N 120.0276° E
Busuanga Island Manganese Mine	Mn	12.1670° N 119.9993° E
Lorraine Orebody–Balabac Island Mine	Al, Fe, Mn, P, SiO_2_	8.0008° N 117.0493° E
Busuanga Island: Coron, Borac-East Mine	Al, Fe, Mn, P, SiO_2_	12.1104° N 119.9993° E
Iron Mining		
Rio tuba Mine	Fe, Ni	8.5868° N 117.4049° E
Lorraine Orebody–Balabac Island Mine	Al, Fe, Mn, P, SiO_2_	8.0008° N 117.0493° E
Busuanga Island: Coron, Borac-East Mine	Al, Fe, Mn, P, SiO_2_	12.1104° N 119.9993° E
Copper Mining		
Balabac Copper Mine in Palawan, Philippines	Cu, Zn	7.9841° N 117.0660° E
Atlas Copper Mine in Palawan	Ni, FeCr_2_O_4_, and other associated mineral deposits	Espanola-9.106383° N, 118.0779211° E, Narra—9.106383° N,118.0779211° E
